# Neoadjuvant tislelizumab plus pazopanib in renal cell carcinoma with venous tumour thrombus: A retrospective study

**DOI:** 10.1002/bco2.70095

**Published:** 2025-11-18

**Authors:** Diliyaer Dilixiati, Houze Li, Huixiang Chen, Shiping Xie, Naifeisha Aihemaiti, Gulizhaer Abulimiti, Baihetiya Azhati

**Affiliations:** ^1^ Department of Urology, Peking Union Medical College Hospital Chinese Academy of Medical Sciences & Peking Union Medical College Beijing China; ^2^ Department of Urology First Affiliated Hospital of Xinjiang Medical University Urumqi China; ^3^ Imaging Center The First Affiliated Hospital of Xinjiang Medical University Urumqi Xinjiang China

**Keywords:** neoadjuvant therapy, pazopanib, renal cell carcinoma, tislelizumab, venous tumour thrombus

## Abstract

**Objectives:**

Neoadjuvant therapy with immune checkpoint inhibitors and tyrosine kinase inhibitors has been shown to reduce the tumour size and thrombus length in patients with renal cell carcinoma with venous tumour thrombus (RCC‐VTT). This study aimed to evaluate the effectiveness and safety of neoadjuvant tislelizumab plus pazopanib in patients with RCC‐VTT.

**Patients and Methods:**

From November 2021 to January 2024, nine patients with RCC‐VTT were included in this retrospective study. All patients received neoadjuvant tislelizumab (200 mg intravenously every 3 weeks) and pazopanib (800 mg orally once daily), followed by surgery. Key effectiveness outcomes included the objective response rate (ORR) of the primary tumour, the percentage change in VTT length and safety.

**Results:**

Among the nine patients with RCC‐VTT, the median age was 58 years (range, 39–78). Following neoadjuvant tislelizumab plus pazopanib, six patients achieved partial response in the primary tumour and three had stable disease, yielding an ORR of 66.7%. The VTT length decreased from 4.9 cm (range, 1.1–9.3 cm) to 3.8 cm (range, 0–8.1 cm), with a median reduction of 29.2% (range, −153.1% to 100.0%). Treatment‐related adverse events (TRAEs) of any grade and grade 3 were reported in 88.9% and 55.6% of patients, respectively. The common TRAEs of any grade were vomiting (77.8%), fatigue (33.3%), pruritus (33.3%), weight loss (33.3%), poor appetite (33.3%), pruritus (33.3%) and hepatic impairment (33.3%). No grade 4–5 TRAEs or deaths were observed.

**Conclusion:**

The neoadjuvant combination of tislelizumab and pazopanib effectively reduced tumour size and thrombus length, narrowing the surgical scope and potentially leading to better postoperative outcomes.

## 1 INTRODUCTION

Renal cell carcinoma (RCC) is the most common type of urogenital cancer, with a mortality rate of 30–40%.^1^ Due to its natural tendency for vascular invasion, 4–15% of patients with RCC are diagnosed with venous tumour thrombus (VTT) involvement, resulting in poor survival outcomes.^2^ Although radical nephrectomy with thrombus removal can improve prognosis, large primary tumour size and extensive thrombus burden may be associated with increased surgical complication rates (up to 70%) and mortality (3–16%).^2^ Therefore, it is crucial to manage the potential complications and mortality by effectively reducing thrombosis and primary tumour size prior to surgery.

Recently, neoadjuvant therapy has emerged as a promising strategy in RCC, improving surgical feasibility and potentially enhancing clinical outcomes.^3^ Within this context, several studies have evaluated neoadjuvant or perioperative single‐agent immune checkpoint inhibitors (ICIs) such as nivolumab in RCC, yielding disappointing outcomes.^4^, ^5^ By contrast, tyrosine kinase inhibitor (TKI) monotherapy has been reported to reduce both thrombus burden and primary tumour size in the neoadjuvant setting.^6^ Karakiewicz et al. initially reported a case of RCC with right atrial thrombus, where neoadjuvant sunitinib led to a reduction in the maximal tumour dimension from 11 to 8 cm, thereby reducing the surgical complexity.^7^ Subsequently, increasing evidence suggests that neoadjuvant TKIs, such as axitinib, sorafenib and pazopanib, can reduce tumour and thrombus burden of RCC‐VTT, potentially improving resectability.^8^, ^9^, ^10^ Given that ICIs combined with TKIs have shown superior efficacy in first‐line treatment of RCC compared with TKI or ICI monotherapy, it has prompted the exploration of this combination regimen in the neoadjuvant setting.^11^, ^12^ Against this background, the NEOTAX trial demonstrated that the combination of toripalimab and axitinib reduced the tumour thrombus length by a median of 2.3 cm in patients with RCC‐VTT.^13^ These findings suggest the feasibility of neoadjuvant ICIs combined with TKIs in this patient population.

Tislelizumab is an anti‐programmed death‐1 (PD‐1) monoclonal antibody with unique structural features and remarkable affinity for PD‐1. Due to its satisfactory anti‐tumour effects, the China National Medical Products Administration has approved tislelizumab for the treatment of several solid tumours and classical Hodgkin lymphoma.^14^ Adding tislelizumab to TKIs such as axitinib, sunitinib and apatinib has demonstrated encouraging efficacy in RCC.^15^, ^16^, ^17^ Moreover, in the neoadjuvant setting, the combination of tislelizumab and axitinib could alleviate the burden of tumour thrombus, enhance the safety and feasibility of surgical resection and improve patient prognosis.^18^ Pazopanib, an orally administered multi‐targeted TKI, has received approvals in America, Europe and several other countries for the first‐line treatment of advanced RCC.^19^, ^20^ Furthermore, a retrospective study has indicated that the combination of pazopanib and ICI was well‐tolerated and could effectively reduce the tumour size and thrombus length in the neoadjuvant treatment of RCC‐VTT.^21^ However, the role of neoadjuvant tislelizumab combined with pazopanib in RCC‐VTT remains unclear. In this study, nine patients with RCC‐VTT were described with the aim of evaluating the effectiveness and safety of neoadjuvant tislelizumab plus pazopanib in this patient population.

## 2 MATERIALS AND METHODS

### 2.1 Patients

This study retrospectively included nine patients with RCC‐VTT who were considered candidates for radical nephrectomy and inferior vena cava thrombectomy and received neoadjuvant tislelizumab plus pazopanib at the First Affiliated Hospital of Xinjiang Medical University from November 2021 to January 2024. Eligible patients were aged ≥18 years, had TNM stage T3‐4N0–1M0–1, a R.E.N.A.L. score of II‐IV, an Eastern Cooperative Oncology Group (ECOG) performance status of 0–1, a life expectancy of at least 3 months and adequate bone marrow, kidney and liver function. Patients who had prior received other anti‐tumour agents, participated in other clinical trials within the past 3 months, had a recent history of cardiac or vascular events, or had contraindications, allergies or adverse events (AEs) to pazopanib or tislelizumab were excluded. This study was approved by the First Affiliated Hospital of Xinjiang Medical University (Ethical Approval Number: K202305–34) and was conducted in accordance with the Declaration of Helsinki and Good Clinical Practice Guidelines. Written informed consent was obtained from each patient.

### 2.2 Treatment regimen

All patients received neoadjuvant therapy with tislelizumab (200 mg intravenously every three weeks) and pazopanib (800 mg orally once daily). Treatment was discontinued prior to the scheduled 12 weeks, and surgery was performed if severe AEs occurred, imaging indicated suboptimal tumour control or at the patient's request. Tislelizumab and pazopanib were discontinued 7 to 14 days before radical nephrectomy or partial nephrectomy.

### 2.3 Outcomes and assessment

The effectiveness outcomes included the ORR of the primary tumour and the percentage change in VTT length. ORR was defined as the proportion of patients who achieved partial response (PR, defined as a 30% decrease in the sum of the longest diameter for all target lesions) and complete response (CR). The primary tumour and VTT size were evaluated by computed tomography (CT) or magnetic resonance imaging (MRI). Tumour response was assessed based on the Response Evaluation Criteria in Solid Tumours (RECIST) version 1.1. CT or MRI scans were performed at baseline, during week 6, and before surgery. Safety was assessed by AEs and surgical complications. The AEs were graded according to the National Cancer Institute Common Terminology Criteria (NCI CTCAE) version 4.0, and the surgical complications were further stratified by the Clavien‐Dindo classification.

### 2.4 Statistics analysis

The statistical analyses were conducted using SPSS version 23.0. Demographic and clinical characteristics were summarized descriptively as median (range) for continuous variables and as number (percentages) for categorical variables. ORR was expressed as a percentage of predicted normal value. The safety analysis was summarized by grade, and the incidence of each event was calculated.

## 3 RESULTS

### 3.1 Patient characteristics

Between November 2021 and January 2024, nine patients (all male) with RCC‐VTT were included in this retrospective study (Tables 1 and 2). The median age was 58 years (range, 39–78), and the majority of patients (66.7%) had an ECOG performance status of 1. All patients were diagnosed with clear cell renal cell carcinoma.

**TABLE 1:** Summary of patient characteristics and clinical outcomes.

Patient ID	Age (years)	Sex	Pathological type	ECOG PS	Clinical stage, TNM	Duration of tislelizumab (cycles)	Duration of pazopanib (weeks)	Length of VTT (cm)			Change of Mayo level	Diameters of primary tumour (cm)			Best response of primary tumour	Surgery
								Baseline	Week 6	Surgery		Baseline	Week 6	Surgery		
1	39	Male	ccRCC	1	T3bN1M0	6	15	6.2	0	0	II → I	8.2	4.9	4.3	PR	Radical nephrectomy
2	78	Male	ccRCC	1	T3bN0M0	2	9	6.7	6.1	5.6	I → I	4.8	3.1	3	PR	Partial nephrectomy
3	62	Male	ccRCC	0	T3cN0M0	4	12	7.7	3.8	3.3	I → 0	11.9	8.2	7.8	PR	Radical nephrectomy
4	62	Male	ccRCC	0	T3bN1M0	4	12	3.2	8.1	8	0 → II	10	5.3	3.5	PR	Radical nephrectomy
5	48	Male	ccRCC	1	T3bN0M0	3	12	1.1	0.7	0.4	0 → 0	6.9	4.8	4.5	PR	Radical nephrectomy
6	71	Male	ccRCC	0	T3aN1M0	3	12	1.3	1.1	1	0 → 0	3.9	2	2	PR	Radical nephrectomy
7	46	Male	ccRCC	1	T3bN0M0	2	9	1.1	0.6	0.6	0 → 0	6.7	5.1	5	SD	Radical nephrectomy
8	58	Male	ccRCC	1	T4N1M1	6	15	9.3	7.1	5.7	I → I	13.4	13	12.7	SD	Partial nephrectomy
9	45	Male	ccRCC	1	T3bN1M0	5	12	7.2	5.1	5	0 → 0	10.3	9.2	9	SD	Partial nephrectomy

ccRCC, clear cell renal cell carcinoma; ECOG PS, Eastern Cooperative Oncology Group performance status; NAT, neoadjuvant therapy; PR, partial response; SD, stable disease; TNM, tumour, node, metastasis; VTT, venous tumour thrombus.

**TABLE 2:** Summary of baseline characteristics of patients.

Characteristics	Patients (n = 9)
Age (years), median (range)	58 (39–78)
Sex, n (%)	
Male	9 (100.0)
ECOG PS, n (%)	
0	3 (33.3)
1	6 (66.7)
IMDC risk score, n (%)	
0–2	6 (66.7)
3	3 (33.3)
Tumour laterality, n (%)	
Right	3 (33.3)
Left	6 (66.7)
Clinical T stage, n (%)	
T3a	1 (11.1)
T3b	6 (66.7)
T3c	1 (11.1)
T4	1 (11.1)
Clinical N stage, n (%)	
N0	4 (44.4)
N1	5 (55.6)
Clinical M stage, n (%)	
M0	8 (88.9)
M1	1 (11.1)

ECOG PS, Eastern Cooperative Oncology Group performance status, IMDC, International Metastatic RCC Database Consortium.

### 3.2 Treatment

As of November 2024, the median follow‐up time was 7 months (range, 5–28 months). The median treatment duration was 4 cycles (range, 2–6) for tislelizumab and 12 weeks (range, 9–15) for pazopanib (Table 1). All patients underwent surgery within 2 weeks following neoadjuvant therapy, with a time interval of 7–14 days. Six (66.7%) patients underwent radical nephrectomy, and three (33.3%) patients received partial nephrectomy. The majority of tumours were removed by laparoscopic nephrectomy (8/9, 88.9%), and 1 (11.1%) was removed via open approach.

### 3.3 Clinical outcomes

**FIGURE 1:**
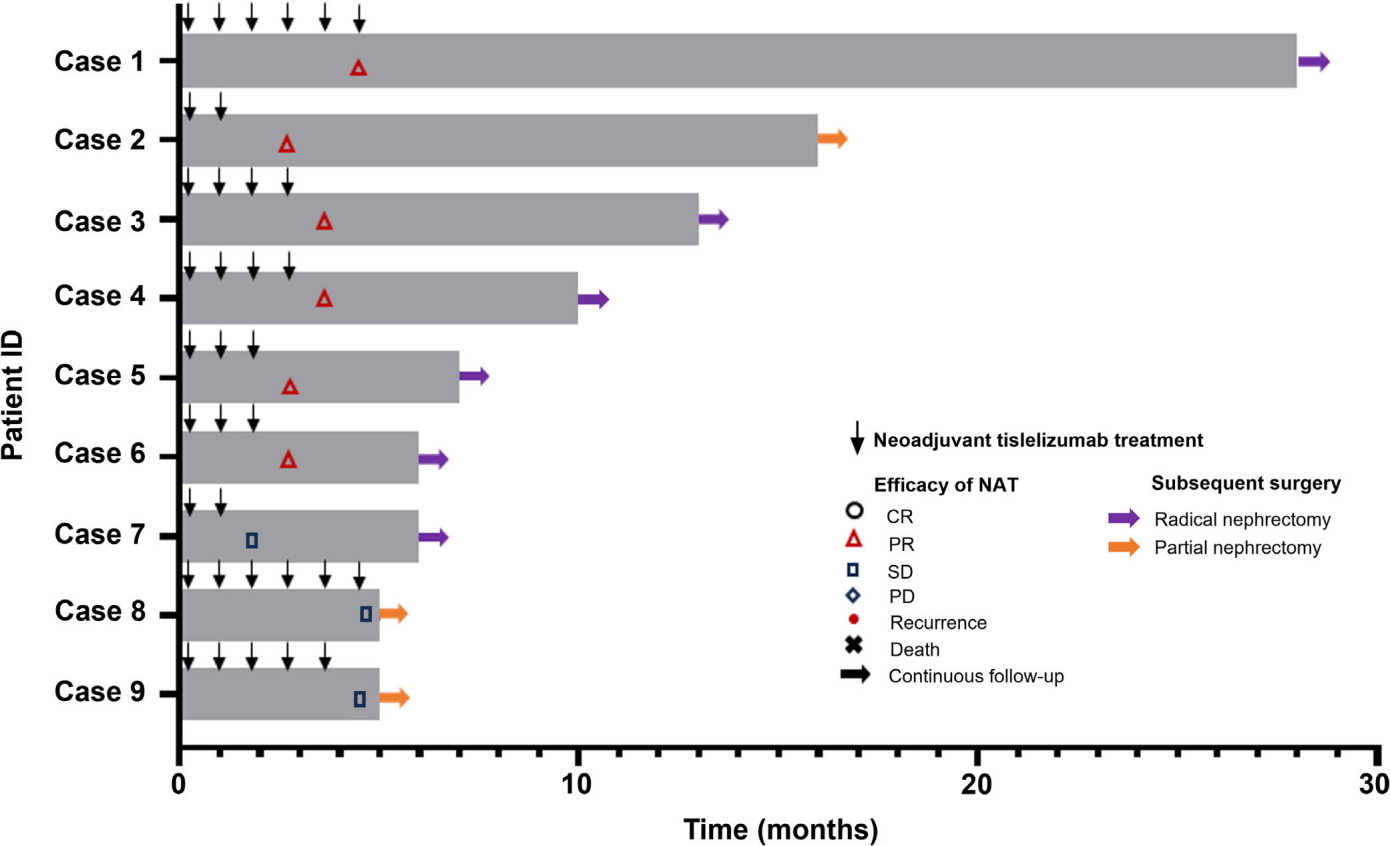
**Swimmer plot of tumour response.** Each bar represents a single patient. CR, complete response; NAT, neoadjuvant therapy; PD, progression disease; PR, partial response; SD, stable disease.

**FIGURE 2:**
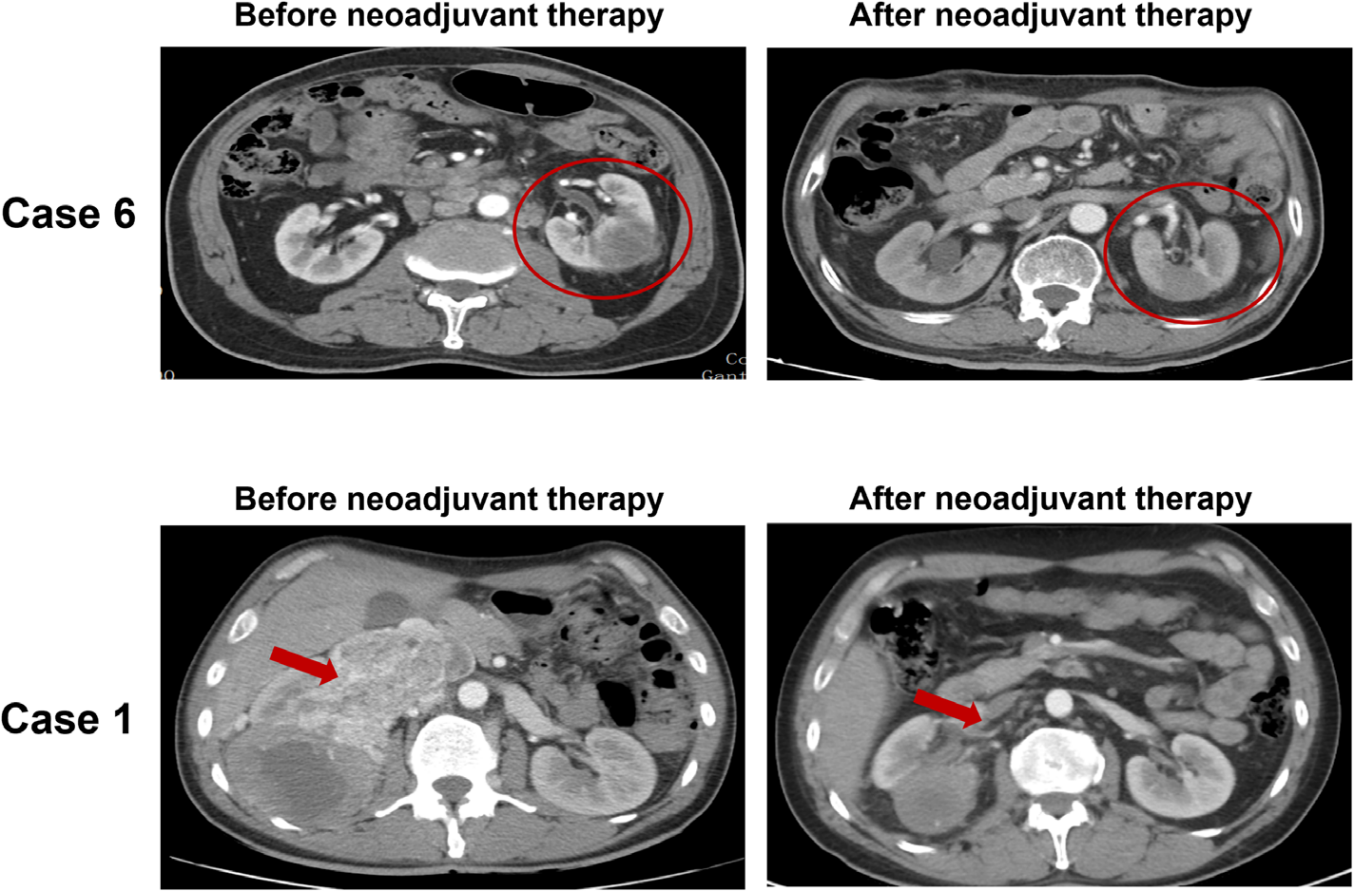
**Changes in tumour thrombus and primary renal tumours in cases 1 and 6.** Left: CT scan or MRI before the neoadjuvant therapy. Right: CT scan or MRI after the neoadjuvant therapy. The red circle indicates the primary renal tumour. The red arrowhead indicates the venous tumour thrombus.

**FIGURE 3:**
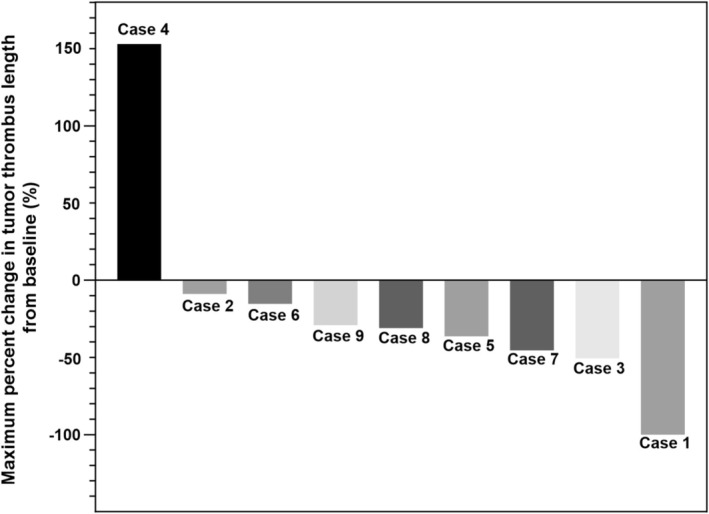
Waterfall plot of maximum percent change in the tumour thrombus length from baseline.

After imaging evaluation, six patients (66.7%) met the RECIST version 1.1 criteria for achieving a PR, while three patients had stable disease (SD), resulting in an ORR of 66.7% (Figures 1 and 2). Following neoadjuvant therapy, the tumour thrombus length decreased from 4.9 cm (range, 1.1–9.3 cm) to 3.8 cm (range, 0–8.1 cm), with a median reduction of 29.2% (range, −153.1% to 100.0%; Figure 3). Only one patient (11.1%) experienced an increase in thrombus length, which rose by 153.1% (absolute value 4.9 cm).

### 3.4 Safety

Of the 9 patients receiving neoadjuvant tislelizumab plus pazopanib, 8 (88.9%) experienced treatment‐related AEs (TRAEs) of any grade (Table 3). The most common TRAEs of any grade were vomiting, which occurred in 7 (77.8%) of 9 patients. Fatigue, pruritus, weight loss, poor appetite, pruritus and hepatic impairment were also common TRAEs, each occurring in 3 (33.3%) patients. Five (55.6%) patients experienced grade 3 TRAEs, including vomiting (4/9, 44.4%), pruritus (2/9, 22.2%) and haematemesis (1/9, 11.1%), with no grade 4 or 5 TRAEs. Five (55.6%) patients discontinued treatment due to TRAEs during neoadjuvant therapy, while none of the reported TRAEs led to dose reduction, surgery delay or death. Postoperatively, two patients experienced Clavien‐Dindo grade ≤2 complications, including constipation (n = 1) and abdominal distension (n = 1), without Clavien‐Dindo grade ≥3 complications observed.

**TABLE 3:** Treatment‐related adverse events after neoadjuvant therapy.

Adverse event, n (%)	Any grade	Grade 3
Total	8 (88.9)	5 (55.6)
Vomiting	7 (77.8)	4 (44.4)
Fatigue	3 (33.3)	0 (0.0)
Weight loss	3 (33.3)	0 (0.0)
Poor appetite	3 (33.3)	0 (0.0)
Pruritus	3 (33.3)	2 (22.2)
Hepatic impairment	3 (33.3)	0 (0.0)
Nausea	2 (22.2)	0 (0.0)
Fever	2 (22.2)	0 (0.0)
Diarrhoea	2 (22.2)	0 (0.0)
Leukopenia	2 (22.2)	0 (0.0)
Haematemesis	1 (11.1)	1 (11.1)
Voice alteration	1 (11.1)	0 (0.0)
Headache	1 (11.1)	0 (0.0)
Cough	1 (11.1)	0 (0.0)
Dizziness	1 (11.1)	0 (0.0)
Jaundice	1 (11.1)	0 (0.0)
Electrolyte imbalance	1 (11.1)	0 (0.0)
Constipation	1 (11.1)	0 (0.0)

## 4 DISCUSSION

To our knowledge, this is the first study to describe the effectiveness and safety of neoadjuvant tislelizumab plus pazopanib in patients with RCC‐VTT. Following neoadjuvant tislelizumab plus pazopanib, the ORR of the primary tumour was 66.7%. Thrombus length was reduced in eight patients (88.9%), with a median reduction of 1.2 cm. No treatment‐related surgical delays were reported. Our findings support the clinical application of neoadjuvant tislelizumab plus pazopanib in RCC‐VTT, which could provide a promising treatment option in this patient population.

Currently, there is no standard neoadjuvant therapy for RCC.^3^ Neoadjuvant TKI monotherapy has shown limited efficacy in RCC with or without VTT, with an ORR of only 15.8–45.8% and tumour thrombus downgrading in approximately 30% of patients.^3^, ^9^, ^22^, ^23^, ^24^ Given that the neoadjuvant combination therapies of ICIs and TKIs have increased the ORR of RCC to 57.1–71.0%, these combinations have been further explored in RCC‐VTT.^18^, ^25^ Recently published studies have demonstrated that neoadjuvant combinations such as avelumab plus axitinib, tislelizumab plus axitinib and toripalimab plus axitinib could reduce tumour thrombus length by 1.5–2.3 cm in patients with RCC‐VTT.^13^, ^18^, ^26^ In the present study, the ORR was 66.7%, and 88.9% of patients receiving neoadjuvant tislelizumab plus pazopanib had a reduction in thrombus length (median reduction, 1.2 cm); both were comparable to the results observed with other neoadjuvant combination regimens. Taken together, these findings indicate that neoadjuvant tislelizumab plus pazopanib exhibits promising antitumor activity and thrombus reduction in RCC‐VTT, supporting its potential as an effective neoadjuvant therapy.

The current safety profile was generally consistent with the AE spectrum of tislelizumab or pazopanib monotherapy.^19^, ^27^, ^28^ The common TRAEs included vomiting, fatigue, pruritus, weight loss, poor appetite, pruritus and hepatic impairment. Although eight (88.9%) patients experienced TRAEs of any grade, the majority were mild, with grade 3 TRAEs occurring in 55.6% of patients, in line with neoadjuvant combination therapies including tislelizumab or pazopanib.^18^, ^21^ Importantly, these events were manageable and tolerable, with no TRAEs leading to treatment discontinuation, surgery delay or death. In terms of postoperative complications, two patients experienced minor postoperative complications (Clavien‐Dindo grade ≤2). No major or newly identified postoperative complications were observed with this neoadjuvant combination, aligning with the results of previous studies.^18^, ^29^ Overall, the safety profile of tislelizumab combined with pazopanib as neoadjuvant therapy was acceptable and well‐tolerated in patients with RCC‐VTT.

Our study had several limitations. First, the selection bias was inevitable due to the single‐arm, single‐centre retrospective design. Second, the study included a limited number of patients, all of whom were from China, which restricts the generalisability of the findings to a broader population. Third, due to the relatively short follow‐up period, data on survival outcomes were still immature at the data cutoff. Finally, exploratory analyses of biomarkers, including systemic inflammatory markers such as C‐reactive protein, were not performed. Therefore, more strictly designed prospective studies with larger sample sizes are needed to verify the efficacy and safety of neoadjuvant tislelizumab plus pazopanib in RCC‐VTT.

## 5 CONCLUSION

In conclusion, this study demonstrated that the neoadjuvant combination of tislelizumab and pazopanib could be effective in shortening tumour thrombus, together with a promising ORR. The safety profile was manageable, with no AEs causing surgical delays. These findings highlight the potential of this neoadjuvant therapy, warranting further investigation to evaluate its potential in treating this challenging disease.

## AUTHOR CONTRIBUTIONS

Diliyaer Dilixiati and Baihetiya Azhati conceived and designed the study; Huixiang Chen recruited patients; Houze Li, Huixiang Chen and Shiping Xie acquired and collected data; Diliyaer Dilixiati, Houze Li, Naifeisha Aihemaiti and Gulizhaer Abulimiti analysed and interpreted the data; Baihetiya Azhati provided administrative support; Diliyaer Dilixiati and Houze Li drafted the manuscript. All the authors have read and approved the final manuscript. Diliyaer Dilixiati and Houze Li have contributed equally to this work and share first authorship.

## CONFLICT OF INTEREST STATEMENT

The authors declare that they have no conflict of interest.

## ETHICAL APPROVAL AND CONSENT TO PARTICIPATE

This study was performed by the Declaration of Helsinki and clinical practice guidelines, and was approved by the ethics committee of First Affiliated Hospital of Xinjiang Medical University (number, K202305–34). Informed consent was obtained from all individual participants included in the study.

## CONSENT FOR PUBLICATION

Not applicable.

## Data Availability

The datasets used and/or analysed during the current study are available from the corresponding author upon reasonable request.
